# HuCoPIA: An Atlas of Human vs. SARS-CoV-2 Interactome and the Comparative Analysis with Other *Coronaviridae* Family Viruses

**DOI:** 10.3390/v15020492

**Published:** 2023-02-10

**Authors:** Naveen Duhan, Rakesh Kaundal

**Affiliations:** 1 Department of Plants, Soils, and Climate/Center for Integrated BioSystems, College of Agriculture and Applied Sciences, Utah State University, Logan, UT 84322, USA; 2 Bioinformatics Facility, Center for Integrated BioSystems, College of Agriculture and Applied Sciences, Utah State University, Logan, UT 84322, USA; 3 Department of Computer Science, College of Science, Utah State University, Logan, UT 84322, USA

**Keywords:** human, SARS-CoV-2, SARS-CoV, MERS, protein-protein interactions

## Abstract

SARS-CoV-2, a novel betacoronavirus strain, has caused a pandemic that has claimed the lives of nearly 6.7M people worldwide. Vaccines and medicines are being developed around the world to reduce the disease spread, fatality rates, and control the new variants. Understanding the protein-protein interaction mechanism of SARS-CoV-2 in humans, and their comparison with the previous SARS-CoV and MERS strains, is crucial for these efforts. These interactions might be used to assess vaccination effectiveness, diagnose exposure, and produce effective biotherapeutics. Here, we present the HuCoPIA database, which contains approximately 100,000 protein-protein interactions between humans and three strains (SARS-CoV-2, SARS-CoV, and MERS) of betacoronavirus. The interactions in the database are divided into common interactions between all three strains and those unique to each strain. It also contains relevant functional annotation information of human proteins. The HuCoPIA database contains SARS-CoV-2 (41,173), SARS-CoV (31,997), and MERS (26,862) interactions, with functional annotation of human proteins like subcellular localization, tissue-expression, KEGG pathways, and Gene ontology information. We believe HuCoPIA will serve as an invaluable resource to diverse experimental biologists, and will help to advance the research in better understanding the mechanism of betacoronaviruses.

## 1. Introduction

In late 2019, cases of pneumonia with an unclear cause occurred in the world. The atypical clinical characteristics were reminiscent of viral pneumonia and triggered worldwide concern due to the severity, quick dissemination, and the potential to affect both the lungs and the possibility of a missed diagnosis [1]. A novel coronavirus [1,2] (nCoV-2019 later renamed to SARS-CoV-2) was found to be associated with these infections. Pathogens emerging and re-emerging are major public health challenges [3]. The *Coronaviridae* family of enveloped RNA viruses has a single-strand genome size ranging from 26 to 32 kilobases, approximately [4]. They are widely spread between humans, other mammals, and birds, causing respiratory, enteric, hepatic, and neurological disorders [5].

Most of these mechanisms involve protein-protein interactions (PPIs). In all living cells, PPIs play a significant role in the infection cycle, as well as in initiating a defense response [6]. Computational methods in particular boost the analysis of host-pathogen protein interactions around the genome [6,7,8,9]. Several data types or characteristics have been developed for the prediction of host-pathogen interactions (HPIs), for example, protein sequence similarity [7], gene ontology (GO) annotations [10], protein dominance interactions [8], and protein structural information [11,12]. Interolog and domain-based techniques for the prediction of inter-species PPIs are widely used [6,13,14,15,16,17]. Many studies have been performed in the past three years to understand the mechanisms of the interactions between SARS-CoV-2 and the human proteome. Most of them concerned SARS-CoV-2’s interactions with human proteins [18,19,20,21,22,23,24,25,26,27]. There is an immediate need to understand the complete PPI network (interactome) between the human proteome and SARS-CoV-2, SARS-CoV and MERS proteins, with localization and functional annotation such as gene ontology, KEGG pathway, tissue expression, which can give insight into pathogenesis and molecular basis of infection [28].

In this study, we examine the use of homology-based (interolog) and domain-based methods to decipher the HPIs between the human and SARS families on a whole-proteome scale. Three different viruses: SARS-CoV-2, SARS-CoV, and MERS, were analyzed against the human proteome. We have compared the common and unique human proteins interacting with these three viruses, and functional characterization of these HPIs may explain the molecular mechanisms for pathogen infections. Through having a detailed functional analysis of the expected interactome to better explain the infections of the illness, we have identified novel protein hubs, enhanced molecular structures, biological processes, tissue expression, and pathways. Furthermore, we have identified the localization of the interacting protein in human cells. Additionally, we have found a difference in HPI patterns between the three viruses. We assume researchers will be able to use the HuCoPIA to search and compare interactions, which could be a good foundation for further experimental validations, and further used in global vaccine development or to provide a deeper understanding of the host of potential virulence factors and drug targets as set out in this analysis. We have compiled all our predictions in a user-friendly database for better visualization.

## 2. Material and Methods

### 2.1. Data Sources

**Figure 1:**
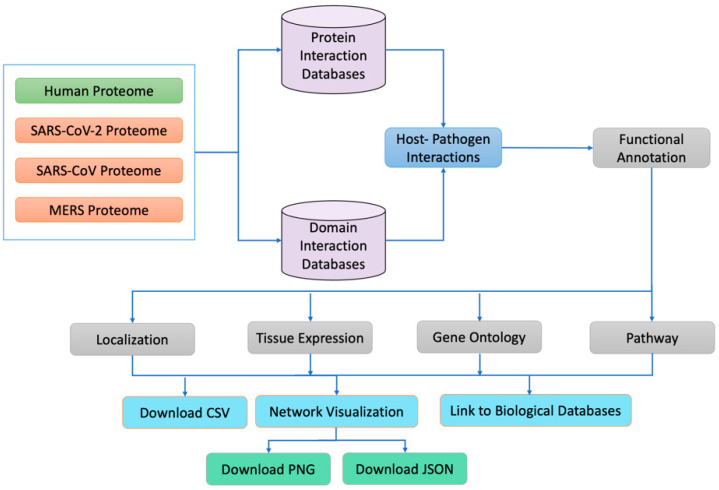
An overall workflow of the HuCoPIA database.

The human reviewed proteome was obtained from (https://www.uniprot.org/facets=reviewed%3Atrue%2Cmodel_organism%3A9606&query=human, accessed on 2 January 2021), SARS-CoV-2 isolates from 30 different countries, 4 isolates of SARS and 1 isolate of MERS were obtained from the NCBI (https://www.ncbi.nlm.nih.gov/labs/virus/vssi/#/virus?SeqType_s=Nucleotide&VirusLineage_ss=SARS-CoV-2,%20taxid:2697049, last accessed on 31 January 2021). The human proteome contains 26,526 proteins, whereas SARS-CoV-2, SARS, and MERS isolates contain 44, 34, and 28 proteins, respectively. We used CD-hit, a very widely used program for clustering and comparing protein or nucleotide sequences, to eliminate duplicate proteins. We considered proteins from different strains to be duplicates if they were 100% identical. Similarly, if they were different, we tagged the protein with the country’s identity. For example, the nsp15 protein is different in an isolate from Iran, so we named the nsp15 of Iran as nsp15_Iran. All human proteins were mapped to their corresponding median tissue expression information, available in the GTEX https://www.gtexportal.org/home/datasets, accessed on 15 July 2021) database. Similarly, the subcellular localizations of the human proteins involved in PPIs were mapped from the Uniport (https://www.uniprot.org/facets=reviewed%3Atrue%2Cmodel_organism%3A9606&query=human, accessed on 12 October 2021) and the Genecard (https://www.genecards.org/Guide/GeneCard#localization, accessed on 12 October 2021) databases. An overall workflow of the HuCoPIA database is depicted in Figure 1.

Several inter- and intra-species interaction databases were used to conduct the interolog and domain-based host pathogen interaction analyses. Six protein-protein interaction databases: HPIDB [29], IntAct [30], DIP [31], MINT [32], BioGRID [33], and HIV [34], have been built up as SQL databases on our own server for Interolog investigation. Similarly, the PFAM [35] database was used to determine the domains present in the human and viral proteomes. IDDI [36], 3DID [37], and DOMINE [38], three domain-domain interaction databases, were downloaded and set up as SQL databases on our server.

### 2.2. Computational Prediction of Host Pathogen Interactions

The interolog-based interaction prediction is based on the conservation of protein-protein interactions across comparable systems. There are no standard cutoff evaluation parameters for defining a true homolog in predicting the HPIs. We found that using an e-value of 1e-50, alignment coverage of 80%, and sequence identity of 40% were the best-fit alignment parameters for predicting the human–betacoronavirus PPIs. Domain-based analysis was one of the computational tools we utilized to uncover the host-pathogen interactions of new COVID-19 strains and the human proteome. The goal of this study is to obtain the domain structures of host and pathogen proteins, then utilize these domain pairings to search DDI databases for probable DDIs. Human proteins are filtered with a 1e-23 e-value and 0.2 coverage, whereas viral proteins are filtered with a 1e-1 e-value and default coverage. To reduce the false positive rate, all projected DDIs are compared to the Negatome database. We use in-house python scripts and queries to link each domain in the DDI to matching proteins. Following the introduction of interolog and domain-domain analysis on an individual basis, it is critical that we combine all the expected host-pathogen interactions into a single set of results. We have combined the data from both methodologies, removed redundant host pathogen interactions, and given more weight to interactions predicted by both interolog and domain-based processes. This is how the PPIs for human and the *coronaviridae* family of viruses have been established. Because we have considered three viruses, we have three consensus files (SARS-CoV-2, SARS, and MERS). Apart from these three, we agreed on the common and different interactions of the three viruses.

### 2.3. Subcellular Localization of Human Proteins Involved in PPIs

Pathogenesis of a wide spectrum of human illnesses is attributed to abnormal protein localization [39]. The understanding of mis-localized proteins might aid in enhancing treatment strategies for a variety of disorders. This information aids pharmaceutical experts in identifying target regions, which aids in the development of new pharmacological treatments and the better utilization of current drugs. Recognizing the relevance of subcellular localization in HPI research, we used the UniProt (https://www.uniprot.org/facets=reviewed%3Atrue%2Cmodel_organism%3A9606&query=human, accessed on 12 October 2021) and Genecard (https://www.genecards.org/Guide/GeneCard#localization, accessed on 12 October 2021) datasets to map the subcellular localization for most proteins. The subcellular localization of all human proteins was downloaded from these two sources and then assigned to the proteins involved in PPIs using our in-house Python scripts.

### 2.4. Tissue Specificity of Human Proteins Involved in PPIs

Genes with disease-related mutations are found in all cells of the body, however, genes that are expressed more heavily in certain tissues can cause functional problems and pathophysiological abnormalities. However, it is critical to comprehend the entire disease-tissue network, since other tissues may be impacted but not show any signs of malfunction. The median tissue expression information was downloaded from the GTEX database [40] (https://www.gtexportal.org/home/datasets, accessed on 15 July 2021). Then, the file was filtered using our in-house Python script and the tissue were selected based on the expression for each protein. Finally, we have highly expressed tissue for each human protein.

### 2.5. Gene Ontology and KEGG Pathways Involved in PPIs

Functional enrichment analysis, Gene Ontology, and KEGG pathway information of the genes were used to provide critical insights on the disease-causing genes and their processes, which is one of the essential phases. To accomplish GO and KEGG enrichment in our investigation, we utilized the R program clusterProfiler [41]. This package was chosen because it used the Benjamini and Hochberg approach for determining the statistical significance of enrichment data [42]. This technique employs an FDR control strategy that reduces not just false positives but also false negatives. We also utilized the cluster profiler package’s simplify function, to get rid of duplicate GO phrases based on semantic similarity measurements. Then the enriched pathways and GO terms were assigned to individual proteins using the in-house Python script.

### 2.6. Implementation

To handle the large PPIs dataset, and for faster results, HuCoPIA has been implemented on a High-Performance Computing cluster; HuCoPIA has been built using MERN stack technology. Express JS and a source-available cross-platform document-oriented database program (MongoDB), classified as a NoSQL database, were used for the back-end development. The React JS application was used to develop the front-end of the database. All the back-end server code, in TypeScript, is available at (https://github.com/usubioinfo/hucobe, accessed on 22 November 2022), and the front-end React JS code can be accessed at (https://github.com/usubioinfo/hucopia, accessed on November 22,, 2022), and finally the database was implemented through PM2, an advanced process manager for JavaScript runtime. The network visualization was implemented using the Cytoscape (https://js.cytoscape.org/, accessed on July 21 March 2022) JavaScript plugin.

## 3. Results

### 3.1. Interolog-Based Interaction Prediction

The interolog-based interaction prediction is based on conservation of protein-protein interactions across comparable systems. For example, if A and A′ are orthologs and B and B’ are orthologs, then the interactions between A and B (in a certain system) and between A′ and B′ (in another system) are interologs [7]. The four proteomes, human, SARS-CoV-2, SARS-CoV, and MERS, were first aligned against each of the six-interaction databases mentioned above, using BLAST’s default parameters. The resulting alignments were filtered with different combinations of e-values (default, 1e-4, 1e-10, 1e-20, 1e-50, 1e-100), sequence coverages (default, 30, 40, 50, 80%), and sequence identities (default, 30, 40, 50, 80%). There are no standard cutoff evaluation parameters for defining a true homolog in predicting the HPIs. However, different cutoff values have been reported by different studies [15,29,43]. Therefore, we used a grid search strategy which generated 150 combinations. In our analysis, we found that using an e-value of 1e-50, alignment coverage of 80%, and sequence identity of 40% were the best-fit alignment parameters for predicting the human–betacoronavirus PPIs. To reduce the false positive PPIs, we have selected a very low e-value and high coverage, because some of the virus proteins were too small. The total predicted interactions by the interolog method are SARS-CoV-2 (95,530), SARS-CoV (93,055), and MERS (55,643). All the following interolog results, predicted PPIs, and functional enrichment analysis are based on these parameters.

### 3.2. Domain-Based Interaction Prediction

Domain-based analysis was one of the computational tools we utilized to uncover the host-pathogen interactions of new COVID-19 strains and the human proteome. The goal of this study was to obtain the domain structures of host and pathogen proteins, then utilize these domain pairings to search DDI databases for probable DDIs. There are a variety of ways for extracting domains from protein sequences; in our case, we used a program called HMMER, which uses hidden Markov models to extract domains from sequence patterns. By comparing our human and viral protein sequences to the HMM-PFAM database, we were able to identify all the domains. The domain pairings between human proteins and viral proteins were then set up in a SQL database named HPD (Human-pathogen domain pairs). To uncover all the potential domain-domain interactions, we brute-forced all the domain pairings in HPD against the three DDI databases (3DID, IDDI, and Domine). The predicted DDIs were then traced back to PPIs using our in-house python scripts and SQL queries to link each domain in the DDI to matching proteins. Several studies have effectively used the profile-HMM technique to predict host-pathogen interactions. Default e-value and coverage settings were used for all protein sequences at first, and then they were filtered to determine the best combination of parameters. Human proteins were filtered with a 1e-23 e-value and 0.2 coverage, whereas viral proteins were filtered with a 1e-1 e-value and default coverage. To reduce the false positive rate, all projected DDIs were compared to the Negatome database. Human and viral protein sequences were aligned with the Negatome dataset first, using severe query settings such as e-values of 1e-3 and 1e-1, identities of 40 and 20, and coverages of 30 and 20 for human and virus proteins, respectively. All the projected erroneous interactions were deleted from the domain-domain analysis predictions. The total predicted interaction by the domain method was SARS-CoV-2 (4296), SARS-CoV (5864), and MERS (6194).

### 3.3. Integration of Results from Two Approaches

Following the introduction of interolog and domain-domain analysis on an individual basis, it was critical that we combined all the expected host-pathogen interactions into a single set of results. To begin, we combined all six interolog result sets (one from each of the six databases), merged them, and deleted duplicate predictions to create a single set of PPIs. Similarly, all three result sets were derived from three DDI databases and blended to retain just the interactions that were similar. We used consensus in the instance of DDA, since the IDDI database projected 30 times more interactions than the other two databases, which is exceedingly unlikely. As a result, we were able to minimize the number of false positives from DDA and arrive at a consistent set of DDIs. In this way we integrated the results from both approaches, deleted the duplicate host pathogen interactions, and given higher weight to the interactions predicted by both interolog and domain-based procedures. This is how the PPIs between human and the *coronaviridae* family of viruses were agreed upon. We have three consensus files since we considered three viruses (SARS-CoV-2, SARS, and MERS). Apart from these three, we reached an agreement on the three viruses’ shared and distinct interactions. Therefore, we have seven sets of consensus results in the conclusion. One set for each of the three viruses, one set for each of the three in which just the interactions unique to that virus are present, and one set in which all three viruses’ interactions are present. The total predicted interactions in consensus between the interolog and domain-based procedures are SARS-CoV-2 (1399), SARS-CoV (1758), and MERS (1002).

### 3.4. HuCoPIA PPI’s Evaluation

For evaluating the predicted PPIs, we downloaded the high confidence, experimentally validated, interactions from [19]. There were 389, 366, and 296 high confidence protein-protein interactions present for SARS-CoV-2, SARS-CoV, and MERS, respectively. We searched these interactions in our PPI data and found 310/389, 291/366, and 276/296 interactions for SARS-CoV-2, SARS-CoV, and MERS, respectively. The detailed experimental interaction results are presented in Supplementary Table S1. Similarly, for the false positive interactions, we downloaded the human-virus negative interactions (70 in total, involving 59 human proteins) from the Negatome [44] and IntAct [30] databases, and searched human protein in our database, finding hits for 19/59 for SARS-CoV-2, 19/59 for SARS-CoV, and 18/59 for MERS, with almost 100 % sensitivity and 76 % specificity. The detailed negative interaction results are presented in Supplementary Table S2. As shown with this example, the chance of getting a false positive interaction is much lower from the database.

## 4. Usage

HuCoPIA can be accessed freely at http://bioinfo.usu.edu/hucopia/ accessed on 22 December 2022. The HuCoPIA database contains SARS-CoV-2 (98,427 (2809 unique interactions)), SARS-CoV (97,161 (1479 unique interactions)), and MERS (60,835 (3408 unique interactions)) interactions. Users can provide a comma-separated list of human genes or upload a text file and select a virus (SARS-CoV-2, SARS-CoV, or MERS), interaction type (Unique, Common, All), interaction type (Interolog, Domain, Consensus), pathogen proteins, and annotation type (Tissue expression, Localization, KEGG pathway, Gene ontology). The resulting interactions can be downloaded in a tab-delimited file. Also, users can visualize the PPI interaction network in a Cytoscape environment with further links to public databases (UniProt, NCBI) for existing annotation details. Users can also download the network as a PNG or in JSON format, which can be opened in network analyzer software such as Gephi, Cytoscape, or others.

## 5. Case Study

**Figure 2:**
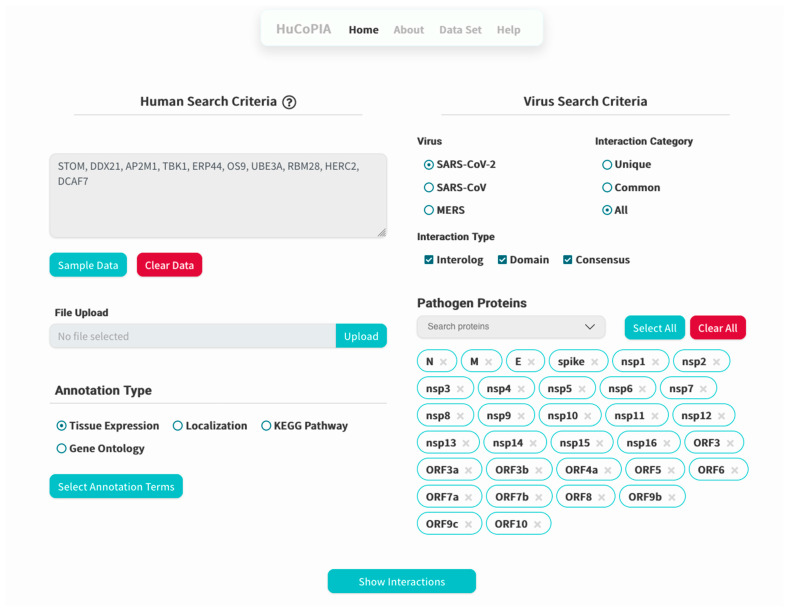
Home page of HuCoPIA.

**Figure 3:**
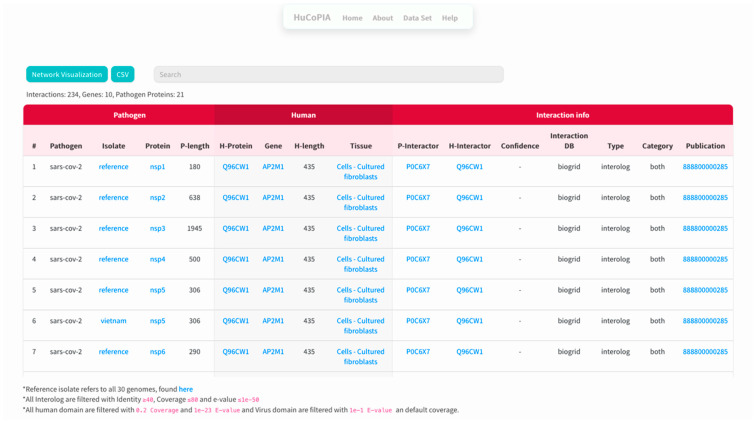
A snapshot of the results page of HuCoPIA.

**Figure 4:**
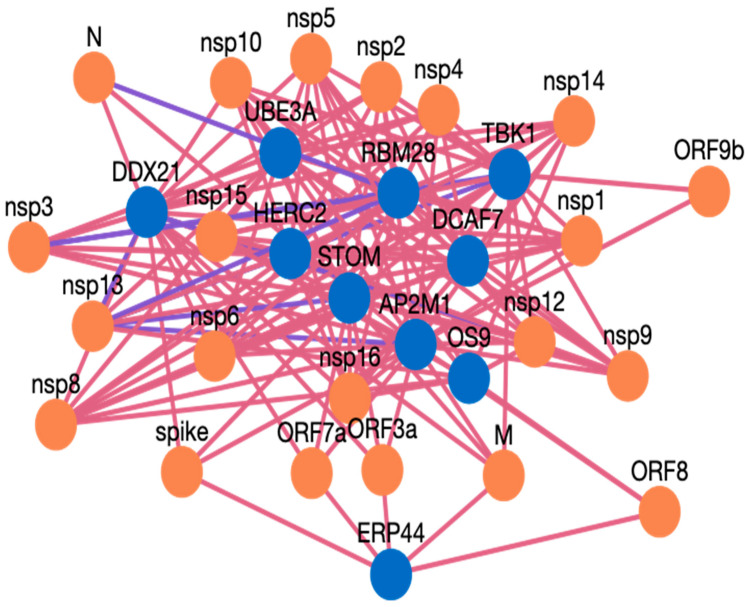
Network visualization of HPIs between ten genes and SARS-CoV-2.

**Figure 5:**
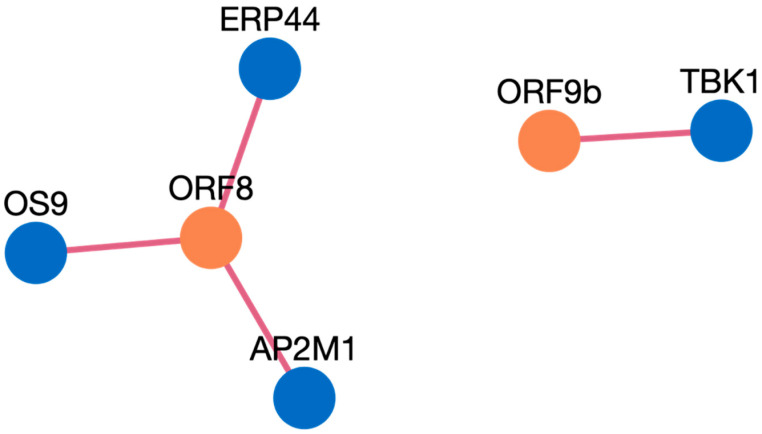
Network visualization of HPIs between ten genes and SARS-CoV.

**Figure 6:**
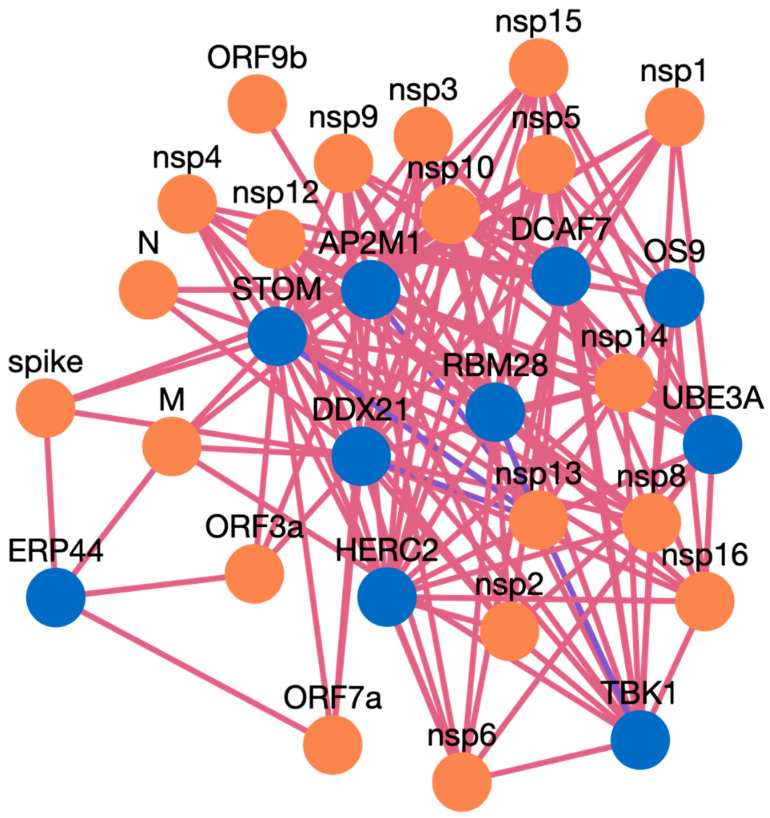
Network visualization of HPIs between ten genes and MERS.

Here, we present an example study on how to search inside the HuCoPIA database. Suppose a user has a list of genes (e.g., STOM, DDX21, AP2M1, TBK1, ERP44, OS9, UBE3A, RBM28, HERC2, DCAF7) from the human genome, and they want to search for interactions based on tissue expression annotation. We have selected these genes involved in virus replication from the literature. The step-wise procedure of searching genes in HuCoPIA is explained in detail below:

1. Upload the genes text file on HuCoPIA (http://bioinfo.usu.edu/hucopia/, last accessed on 22 December 2022), select all virus proteins from the dropdown for each virus, and select tissue expression as the annotation type. Figure 2 shows the homepage of HuCoPIA with the search options. An intermediate results page will be shown after ‘show interactions’ page.
2. The search results are displayed in a table. Information such as pathogen protein, pathogen isolate, pathogen protein length, human gene, human protein, human protein length, functional annotation, interaction source database, confidence, and PubMed links are available. Figure 3 depicts the results page. Further, a user can download the results as a csv file or visualize them in a network. Users can click on the proteins, or other links in blue, to check the detailed information. For the ten genes we submitted, there are 234 interactions involving 21 virus proteins. The results are presented in Supplementary Table S3.
3. The network visualization of the interactions is generated by clicking on the network visualization button, and the resulting network is displayed with a table. In the info section, users can get information about the genes. The network is displayed in Figure 4, Figure 5 and Figure 6.

### Case Study Genes Discussion

AP2M1 is reported to be a crucial host factor for SARS-CoV infection. Based on the previously reported discovery of sunitinib, a kinase inhibitor is involved in the regulation of AP2M1. AP2M1 encodes the μ2 component of the AP2 complex, which is a clathrin adapter protein complex. AP2M1, clathrin, and other components form a clathrin-dependent endocytic pathway by which cells absorb metabolites, hormones, proteins, and viruses via inward budding of the plasma membrane [45,46]. In our study, we found that AP2M1 interacts with 21 SARS-CoV-2 proteins, one SARS-CoV-2 protein, and 20 MERS proteins. The DDX DEAD box RNA helicase is a versatile protein that is involved in every step of RNA metabolism. As a result, host RNA helicases may be able to govern and maintain a huge viral RNA genome. DDX21 has been reported to strongly restrict the entry of SARS-CoV-2, while others such as DDX1, DDX3, DDX5, and DDX6 were required for SARS-CoV-2 replication. The SARS-CoV-2 N protein interacts with DDX6 and hijacks it to carry out viral replication [47]. We found that DDX21 interacts with 19 SARS-CoV-2 proteins, no SARS-CoV proteins, and 19 MERS proteins. Heterozygous detrimental mutations in the TBK1 gene have been linked to severe COVID-19 infection [48]. TBK1 was found to interact with 16 SARS-CoV-2 proteins, one SARS-CoV protein, and 15 MERS proteins. ER stress and UPR induction activation by SARS-CoV-2, and SARS-CoV ORF proteins has been reported [49]. The ERP44 protein interacts with five SARS-CoV-2 proteins, one SARS-CoV proteins, and four MERS proteins. RBM28 and other RNA binding proteins have been reported to interact with the SARS-CoV-2 interactome [50]. ER-associated degradation pathways are involved in MHV-induced DMV formation and viral replication, and effectors like OS9 are involved in this [51,52]. OS 9 was found to interact with nine SARS-CoV-2 proteins, one SARS-CoV proteins, and eight MERS proteins. STOM has also been reported to interact with SARS-CoV-2 proteins [53], and we found it interacting with 18 SARS-CoV-2, and 18 MERS proteins, while for SARS-CoV there was no interaction predicted. The interaction of ubiquitin and proteasome is also required for the various phases of the coronavirus infection cycle [54]. HERC2 was found to interact with 14 SARS-CoV-2 proteins and 14 MERS proteins, but no PPI were obtained for SARS-CoV. The UBE3A protein is reported to be cleaved by the SARS-C-V-2 Mpro protein [54,55]. In our study, we found UBE3A to interact with 14 SARS-CoV-2 and 14 MERS proteins. DCAF7 has also been reported as a drug target for COVID 19 [56]. DCAF7 is also found to interact with 14 SARS-CoV-2 and MERS proteins. We believe that these example proteins from the case study are all good candidates for drug targeting, and that these protein interactions could be further validated for use as drug targets against these three viruses. Similarly, HuCoPIA can be used to explore other novel targets in humans against these three *coronaviridae* family viruses.

## 6. Conclusions

HuCoPIA is a database of computationally identified protein-protein interactions between the human proteome and three strains (SARS-CoV-2, SARS-CoV, and MERS) of betacoronavirus. HuCoPIA provides a user-friendly interface where users can search for protein-protein interactions by selecting different options such as common or unique interactions across the three viruses, and different functional annotations. It provides access to functional annotations of the human proteome, for example, gene ontology, tissue expression, localization, and KEGG pathway. The comparison of these viruses allows us to obtain common or unique interactions between human proteins and these viruses. Moreover, it provides the most highly expressed tissues and subcellular localization in the human genome. Interactions present in HuCOPIA are computationally predicted, and though we have removed the false interactions with the Negatome database, still these interactions need further experimental validation. We will keep updating the protein functional annotation information inside HuCoPIA as per the availability in biological databases, and remove more false positives based on the availability of negative interactions. Researchers will be able to use HuCoPIA to search and compare interactions and then these interactions can be validated experimentally and further used in global (e.g., pan viral) vaccine development or to better understand the pathogenesis mechanisms.

## Data Availability

All the predicted interactions and visualizations are available inside the database which can be freely accessed at http://bioinfo.usu.edu/hucopia/ (accessed on 7 November 2022).

## Supplementary Materials

The following supporting information can be downloaded at: https://www.mdpi.com/article/10.3390/v15020492/s1.
